# Intersectional discrimination and mental health in later life: ageism as a core dimension

**DOI:** 10.1093/geronb/gbaf184

**Published:** 2025-09-24

**Authors:** Yi Wang, Yifan Lou, Huei-Wern Shen, Ernest Gonzales

**Affiliations:** School of Social Work, University of Iowa, Iowa City, Iowa, United States; School of Social Work, Virginia Commonwealth University, Richmond, Virginia, United States; Department of Social Work, College of Health & Public Service, University of North Texas, Denton, Texas, United States; Silver School of Social Work and Center for Health and Aging Innovation, New York University, New York City, New York, United States

**Keywords:** Bias, Cognitive functioning, Depression, Loneliness

## Abstract

**Objectives:**

Despite extensive literature that examines the relationship between discrimination and health, less is known about specific discrimination attributions and how they may differentially associate with health. To address this gap, the current study investigated the latent typology of discrimination attributions and the intersectional attributions’ relationships with mental health in later life.

**Methods:**

Data came from 6,282 respondents in the 2016 Psychosocial Leave-Behind Questionnaire of the Health and Retirement Study. Participants ascribed their everyday discrimination experiences to a list of potential reasons (e.g., ethnicity, ancestry, gender, race, age, religion, and financial). Latent class analysis was performed to identify discrimination attribution typologies. Regression models with marginal effects were conducted to explore differential health associations of different attribution typologies.

**Results:**

Five distinct typologies were identified: few discrimination experiences (33%), discrimination with no specified attributes (5%), discrimination due to age (48%), discrimination due to age, race, and ethnicity (8%), and discrimination due to age, explicit physical characteristics, and socioeconomic disadvantages (5%). Regression analysis revealed significant associations between discrimination and mental health indicators such as depressive symptoms and loneliness. Discrimination involving more than just age, especially physical and socioeconomic disadvantages, had strong negative associations with health.

**Discussion:**

Ageism emerged as a core dimension and prevalent theme and often co-occurs with other characteristics, highlighting the intersectionality of perceived discrimination. The negative health associations were most pronounced for those who experienced discrimination related to intersectional attributions. Implications for social policies, practice, and research were discussed.

## Discrimination is prevalent and harmful

Discrimination, defined as the behavioral expression of negative evaluations toward individuals based on their membership in a particular group (Allport, 1958; Stuber et al., 2008), is prevalent among older adults in the United States. Studies have consistently shown that approximately 60% of older adults report being a target of day-to-day discrimination, and one-third report at least one major discriminatory event during their lifetime (Kessler et al., 1999; Luo et al., 2012). Beyond its prevalence, discrimination has also been recognized as a significant risk factor contributing to various health issues, including high blood pressure, breast cancer, and ultimately, elevated mortality rates (e.g., Cobb et al., 2022; Farmer et al., 2019; Sims et al., 2022; Taylor et al., 2007). Furthermore, perceived discrimination has been associated with a range of mental health issues, including depression, psychological distress, cognitive impairment, and anxiety (e.g., Moody, 2022; Williams & Mohammed, 2009; Zahodne et al., 2020).

## Discrimination as a predictor of health: theory and evidence

The fundamental premise of research examining the discrimination–health association is that discriminatory experiences influence health outcomes through the stress responses they elicit (Pascoe & Smart Richman, 2009). Many studies, both experimental and non-experimental, draw on theoretical models that conceptualize discrimination as a potent source of stress for marginalized groups, such as the stress process model (Pearlin et al., 1981; Turner, 2009), the social stress theory (Aneshensel, 1992), and the minority stress framework ­(Forrester et al., 2019). These frameworks highlight how stress exposure and access to coping resources are shaped by social status (e.g., race, ethnicity, and income). Individuals in lower social status groups encounter discrimination more frequently and, unfortunately, often possess fewer resources to buffer or mitigate the effects of stress (Perry et al., 2013). Discrimination is particularly detrimental to health, as it not only compounds general life stressors but also imposes the unique burden of discriminatory bias and social exclusion. Consequently, the health impacts of discrimination tend to be more pronounced for individuals in lower social status groups, thereby exacerbating existing health disparities.

Empirically, discrimination has been examined as a stressor with significant psychological and physiological consequences (Sawyer et al., 2012). The literature suggests that perceived discrimination is related to heightened physiological stress responses, including dysregulation and overaction of physiological regulatory systems. Chronic exposure to discrimination can undermine self-esteem and feelings of control, further elevating the risk of developing mood and anxiety disorders (Gee et al., 2007). Additionally, experiencing discrimination may increase the likelihood of engaging in unhealthy behaviors or reduce participation in healthy behaviors, both of which may serve as short-term, adaptive stress-reducing coping mechanisms (Jackson et al., 2002; Pascoe & Smart Richman, 2009). These pathways can lead to significant physiological deterioration at midlife and older ages, ultimately contributing to an increased risk of morbidity and mortality (Jackson et al., 2011; Williams & Mohammed, 2009). Studies have also documented discrimination as a critical determinant driving health disparities, including hypertension, chronic inflammation, and psychiatric disorders, particularly among older Black adults (Beatty et al., 2014; Lewis et al., 2009; Mouzon et al., 2017).

## The intersectionality of discrimination

The concept of “intersectionality,” first introduced by African-American scholar Kimberlé Crenshaw (1989), emphasizes that individuals can experience multiple, overlapping forms of discrimination. Unlike the unitary approach, which focuses on a single category of social position, the intersectional approach considers how multiple, intersecting social identities shape lived experiences (Gendron et al., 2024; Hancock, 2007). For example, while white women may face gender discrimination, racial and ethnic minority women are often subject to both gender and racial discrimination. Importantly, these experiences of multiple subordination cannot be simply reduced to the sum of each type of discrimination (Bauer, 2014). To fully understand the discrimination experienced by Black women and men, for instance, it is necessary to move beyond treating gender and race as separate factors and consider other dimensions, such as socioeconomic status, sexuality, and nativity, simultaneously to fully understand the complex intersecting effects of discrimination on health outcomes. This approach acknowledges that societal experiences vary significantly based on how these identities intersect (Bauer & Scheim, 2019; ­Ferraro & Zaborenko, 2023). An intersectional approach is essential for all societal groups, as no single characteristic can fully capture an individual’s lived experience of discrimination (Bowleg, 2012; Gendron et al., 2024; Ferraro & Zaborenko, 2023). Prior literature also suggests that specific combinations of discrimination, particularly ageism alone or in conjunction with other biases like racism, ethnicism, ableism, or classism, are common among older adults. For instance, studies using the Health and Retirement Study (HRS) indicate that about one-third of individuals aged 50 and older reported ageism as the primary reason for discrimination, with ageism frequently co-occurring with racism and ethnicism among minority groups (Ferraro & Zaborenko, 2023; Luo et al., 2012). Another study discovered middle-aged women who experienced cumulative perceived interpersonal discrimination had the highest risk of depression, relative to women in other latent class profiles (Bécares & Zhang, 2018). Notably, this risk was consistent across race and ethnicity, meaning all women, regardless of race and ethnicity, experienced high rates of depression due to cumulative interpersonal discrimination. Additional research also highlights the intersections of ageism with ableism or classism, underscoring the prevalence of these overlapping discrimination experiences (Bauer & Scheim, 2019).

Scientific inquiry into the relationship between discrimination and health must account for the interlocking effects of social stratification and multiple types of discrimination (Bécares & Zhang, 2018; Ferraro & Zaborenko, 2023), such as racism, ableism, and ageism, to better understand how these overlapping systems of oppression associate with health. Intersectional discrimination can contribute to stress later in life through various mechanisms, including diminished income-generating capacity, reduced power, and an increased burden of comorbidities and frailty (Sims et al., 2022). Compared to discrimination ascribed to a single attribute, discrimination stemming from multiple aspects of social constructions of identity may deprive individuals of greater socioeconomic and psychological resources, potentially leading to unhealthy behaviors and worse health outcomes (Bauer & Scheim, 2019).

## Gaps in literature

Despite the well-documented relationship between discrimination and health, much of this literature tends to focus solely on the *frequency* of perceived everyday discrimination, such as how often individuals are threatened, harassed, treated with less respect, receive poorer service, or are regarded as unintelligent. The response options typically range from “never” to “less than once a year,” to “at least once a week,” and “almost every day” (e.g., Farmer et al., 2019; Pascoe & Smart Richman, 2009). However, this approach often overlooks the specific types and underlying reasons for discrimination (i.e., attribution; Ferraro & Zaborenko, 2023). Among the scant research that delved into the discrimination attributions, most studies remained focused primarily on *singular* forms of discrimination involving race, ethnicity, indigenous status, immigrant status, age, or sexuality (e.g., Allen et al., 2022; Kaholokula et al., 2012; Klonoff et al., 2000; Ryan et al., 2006; Williams & Mohammed, 2009), without adequately addressing the complex nature of co-occurring discriminatory experiences.

Given its high prevalence, wide distribution, and strong links to health, perceived discrimination warrants deeper investigation, particularly regarding the intersectionality of its sources to inform public policies and programs. To our knowledge, only a limited number of studies have explored multiple attributions for discrimination. Some studies (Cobb et al., 2022; Sims et al., 2022) have operationalized “intersectional discrimination” by simply counting the number of attributed reasons for perceived everyday discrimination (e.g., ascribing discrimination to three or more reasons, two reasons, or no more than one reason) or utilizing latent class analyses among middle-aged women (Bécares & Zhang, 2018). These approaches do not adequately capture the underlying patterns and structures of the discrimination types in later life, as discrimination often arises from intersecting social identities—such as age, race, gender, class, and physical ability—whose impact can profoundly shape health. This is especially relevant for older adults, who may be particularly vulnerable to the compounded and cumulative effects of various forms of bias.

## The present study

To address these gaps, this study aims to investigate typologies of perceived interpersonal discrimination using latent class analysis (LCA), a novel approach for this context. LCA can help identify qualitatively distinct subgroups within a population by creating typologies (classes) based on the attributed reasons for everyday discrimination. This study seeks to explore older adults’ self-reported discrimination attributions and the nuanced ways in which different sources of discrimination relate to mental health indicated by depressive symptoms and loneliness. The findings will provide insights into the prevalence of specific typologies of discrimination attributions and reveal which patterns are most strongly associated with adverse mental health outcomes.

## Methods

### Data and sample

The study uses data from the HRS, conducted by the Institute for Social Research at the University of Michigan. HRS is a nationally representative biennial survey of U.S. adults aged 50 and older, examining various aspects of aging, including socioeconomic status, health and mental health, labor participation, and household structures. HRS follows the respondents over the years once recruited and replenishes the sample every 6 years with a younger cohort to ensure sample representativeness (Sonnega et al., 2014). Initially piloted in 2004 and formally launched in 2006, HRS administered a self-report mail-in psychosocial survey, known as the “leave-behind questionnaire” (LBQ). Various topics were included in LBQ, such as life satisfaction, perceived social support, and experiences and attributions of everyday discrimination (Fisher & Ryan, 2018).

Although the HRS has released the 2022 full-sample dataset, it is currently in early-release form. Therefore, the present study draws on data from the 2016 HRS dataset, the most recent wave with replenished samples, ensuring data representativeness. Among the 20,912 respondents in the 2016 HRS, approximately half of the participants received the LBQ upon the completion of the in-person core survey, with 6,369 LBQ surveys returned by mail. Our analytic sample includes 6,281 participants who responded to discrimination-related questions.

### Measures

#### Attribution for everyday discrimination

We included 1 dichotomous indicator for whether participants experienced any type of discrimination (0 = never experienced) and 11 indicators for reasons for discrimination in the LCA. Those who responded experiencing any discrimination could then specify the reasons for these experiences by selecting from 11 possible attributions: ancestry/ethnicity, gender, race, age, religion, weight, physical disability, aspects of physical appearance, sexual orientation, financial status, or other. Participants were allowed to select multiple attributions that applied to their experiences. Notably, approximately 3%–4% of participants did not report the intensity of everyday discrimination but provided reason(s) during the follow-up questions. These cases were retained to capture the full range of attribution patterns, as they likely reflect experiences of subtle discrimination not detected by the initial intensity question (e.g., daily or less than once a year). Excluding them could bias the LCA by underrepresenting certain typologies.

#### Mental health

Two mental health outcomes of interest were examined: depressive symptoms and loneliness. Depressive symptoms were measured using the eight-item version of the Center for Epidemiologic Studies Depression Scale (Radloff, 1977), which includes symptoms such as feelings of sadness, loneliness, and restless sleep. Two items (“I enjoyed life” and “I felt hopeful about the future”) were reverse-coded. Responses are averaged, yielding scores from 0 to 8, with higher scores indicating more depressive symptoms (α = 0.79). Loneliness was measured with the 11-item UCLA Loneliness Scale. Respondents rate the frequency of each item on a scale from 1 (*often*) to 3 (*hardly ever or never*). Example items include “Isolated from others” and “There are people you can turn to.” Some items were reverse-coded so that higher scores consistently reflect greater loneliness. The individual response scores were then averaged. Cases with more than five missing items were assigned as missing, following the RAND’s approach (Bugliari et al., 2019).

#### Covariates

We included sociodemographic and health-related covariates that may confound the relationship between everyday discrimination attributions and health, as suggested by previous literature (Cobb et al., 2023; Jackson et al., 2002; Perry et al., 2013). Sociodemographic variables included age (1 = 50–64, 2 = 65–74, 3 = 75+), gender (1 = female), race/ethnicity (1 = non-Hispanic White, 2 = non-Hispanic Black, 3 = Hispanic, 4 = Others), marital status (1 = married), years of education (1–17), household annual income (0 = $0–$20,000; 1 = $20,001–$40,000; 2 = $40,001–$60,000; 3 = $60,001–$100,000; 4 = $100,001+), urban residency (1 = urban resident), and U.S. birth status (1 = born in the United States). Health covariates included self-rated health (from 1 = poor to 5 = excellent); the number of difficulties in activities of daily living (ADLs; 0–6), such as bathing, dressing, eating, getting in and out of bed, walking, and toileting; the number of difficulties in instrumental activities of daily living (IADLs; 0–4), including using the phone, taking medications, shopping for groceries, and preparing hot meals; and neuroticism, assessed using the four-item neuroticism subscale from the Midlife Development Inventory (Lachman & Weaver, 1997), which includes moody, worrying, nervous, and calm, rated on a four-point scale (1 = not at all, 4 = a lot). Three items (moody, worrying, and nervous) were reverse-coded so that higher values indicate higher levels of these traits. Responses were then averaged to create a composite neuroticism score (range: 1–4), with higher scores indicating greater neuroticism (Cronbach’s α = 0.70).

### Analytical steps

We first used LCA to identify distinct typologies of discrimination attributions among the 6,281 respondents, based on 1 dichotomous measure of whether they experienced any type of discrimination and 11 discrimination attributions described in the Measures section. LCA identifies unobserved subgroups by analyzing patterns in respondents’ responses to co-occurring conditions (Nylund-Gibson et al., 2023). We fitted LCA models with 2–6 classes to select the best model based on both model fit statistics and the interpretability of the classes. Model fit statistics included Akaike’s Information Criterion (AIC), Bayesian Information Criterion (BIC), and entropy. Lower AIC and BIC values indicate better model fits, while higher entropy reflects better classification quality (Nylund et al., 2007). To determine the optimal number of classes, we also used diagnostic tools for classification precision, such as Model Class Proportion (mcaP), Average Posterior Probability (AvePP), and Odds of Correct Classification (OCC). mcaP measures the relative size of each class assignment, with smaller differences between mcaP and model-estimated proportions indicating less classification error (Nylund-Gibson et al., 2023). AvePP, ranging from 0 to 1, represents the proportion of samples in each class correctly classified. A higher AvePP indicates better accuracy, with 0.7 being a common threshold for adequate performance (Nylund et al., 2007). OCC measures the odds of model-estimated class assignment relative to random assignment based on class proportion. A higher OCC indicates better precision, with values above 5 indicating superior precision (Nylund et al., 2007). The LCA was conducted in MPLUS with a *mixture* statement. The LCA model estimates parameters using all available information and accounts for missingness under the assumption of Full Information Maximum Likelihood. In our analysis, all observations used in the LCA are complete cases.

After determining the optimal number of classes, each participant was assigned to the class for which they had the highest probability of membership, using the maximum likelihood estimation. We then applied two separate multivariate linear regression models (Models 1 and 2) to associate each of the mental health domains (depressive symptoms and loneliness) using the predicted class membership from the LCA as the independent variable, controlling for sociodemographic and health-related covariates. Following the regression, we calculated the marginal effects at the mean of each health outcome by latent class membership. Cases with missing data (<5%) in the regression analysis were handled using listwise deletion. An examination of missing data patterns revealed no systematic differences in key variables, suggesting negligible risk of bias. The regression models and post-estimation margins were conducted in STATA 17.0.

## Results

### Latent class analysis

The model fit for the two- to six-class solutions is presented in Table 1. BIC values successively decreased with each additional latent class, indicating relative improvements in the model fit, up to the six-class solution. However, the entropy of the six-class model decreased compared to the five-class model, indicating a reduced classification quality and suggesting that the five-class model provides the most accurate classification. The five-class model demonstrated strong classification accuracy, with minimal differences between the model-estimated proportion and mcaP. Additionally, AvePP values were consistently above 0.7 across all classes, and OCC values were high, exceeding 5. Moreover, the six-class solution included at least one latent class with less than 5% prevalence, and some of its classes were conceptually similar to others, lacking distinctiveness. Therefore, we opted for the more parsimonious five-class model (Table 2).

**Table 1.** Results of latent class analysis (*n *= 6,281).

Model	*df*	AIC	BIC	Entropy	Note
**Two-class model**	4035	47474.283	47642.919	0.798	
**Three-class model**	4045	46153.503	46409.830	0.819	
**Four-class model**	4029	45597.031	45941.049	0.804	
**Five-class model**	4021	45196.345	45628.053	0.874	Selected
**Six-class model**	4007	44987.706	45507.105	0.864	

*Note*. AIC = Akaike’s Information Criterion; BIC = Bayesian Information Criterion. Model fit statistics were used to determine the optimal number of classes.

**Table 2.** Five-class model classification accuracy and item-response probabilities.

Variable	Class 1: Few discrimination experiences (33%)	Class 2: No specified attributes (5%)	Class 3: Ageism (48%)	Class 4: Ageism + racism and ethnicism (8%)	Class 5: Ageism + physical and socioeconomic characteristics (5%)
**Classification accuracy^a^**					
**Modal Class Assignment Proportion (mcaP)**	0.33	0.06	0.50	0.07	0.04
** Average Posterior Probabilities (AvePP)**	0.98	0.92	0.92	0.82	0.84
** Odds of Correct Classification (OCC)**	99.75	199.70	12.61	52.80	89.10
**Item-Response Probabilities^b^**					
** Have experienced discrimination**	0.02	1.00	0.96	0.96	0.88
** Reason: ethnicity**	0.00	0.00	0.04	**0.66**	0.26
** Reason: gender**	0.00	0.02	0.14	0.43	0.38
** Reason: race**	0.00	0.00	0.10	**0.95**	0.33
** Reason: age**	0.00	0.01	**0.44**	**0.43**	**0.76**
** Reason: religion**	0.00	0.00	0.04	0.16	0.27
** Reason: weight**	0.00	0.00	0.12	0.12	**0.68**
** Reason: disability**	0.00	0.00	0.09	0.05	**0.63**
** Reason: appearance**	0.00	0.02	0.12	0.15	**0.63**
** Reason: sexual**	0.00	0.00	0.01	0.05	0.20
** Reason: financial**	0.00	0.01	0.12	0.34	**0.66**
** Reason: other**	0.01	**1.00**	0.03	0.03	0.04

a Values reported for each class in classification accuracy represent the model-estimated proportion.

b Item-response probabilities represent the likelihood that respondents within a given class will endorse each question; the item-response probabilities in bold indicate the highest probabilities of reporting specific attributions within the latent class and/or across classes.

Based on the overall response probability patterns, the five classes were labeled as follows: C1: *few discrimination experiences* (33%), characterized by the lowest likelihood of experiencing any form of discrimination; C2: *discrimination with no specified attribution* (5%), characterized by the absence of identified reasons for discrimination; C3: *discrimination due to their age* (48%), characterized by attributing discrimination mainly to ageism; C4: *discrimination due to age*, *race*, and *ethnicity* (8%), characterized by recognizing ageism, racism, and ethnicism as the predominant reasons for discrimination; and C5: *discrimination due to age*, *explicit physical characteristics*, and *socioeconomic disadvantages* (5%), linked with weight, physical disability, appearance, or financial status, in addition to ageism. Notably, approximately 15% (*n *= 40) of participants in C5 did not report the intensity of discrimination but provided an attribution in the follow-up, while this rate is about 4% in other groups.

### Sample characteristics and group comparisons

Table 3 summarizes the sample characteristics in the total sample and by latent classes. Close to half (45.69%) of the respondents were under 65 years old, 24.61% young-old (65–74), and 29.70% aged 75+. The sample was predominantly non-Hispanic White (64.18%), followed by non-Hispanic Black (18.26%), Hispanic (13.39%), and other (4.17%). The majority were women (60.04%), married (57.45%), urban residents (53.07%), and U.S.-born (86.61%). The average years of education was 12.78 (*SD* = 3.26). Approximately 45% of participants reported a yearly household income of less than $40,000, while over 20% reported an income exceeding $100,000. As for self-rated health status, the average rating was 3.05 (*SD* = 1.07), an equivalent of “Good” on the scale. This sample reported an average neuroticism score of 1.97 (*SD* = 0.62), an average of 0.59 (*SD* = 1.31) difficulties in ADLs, and 0.28 (*SD* = 0.79) difficulties in IADLs. The mean scores for depressive symptoms and loneliness were 1.54 (*SD* = 2.03) and 1.55 (*SD* = 0.44), respectively.

**Table 3.** Characteristics of individuals in each latent class (*n *= 6,281).

Characteristics	Total	C1	C2	C3	C4	C5
**Observations, *n***	6,281	2,071	351	3,113	466	280
**Proportions, %**	100	32.95	5.64	49.57	7.42	4.46
**Have experienced discrimination, %**	64.73	0.00	100.00	97.21	96.35	85.71
**Demographics**						
Age, %*** ^,^ ^a^ ^,^ ^b^ ^,^ ^c^						
** 50–64**	45.69	35.74	66.47	42.58	67.1	56.99
** 65–74**	24.61	26.9	19.46	25.55	19.56	20.59
** ≥75**	29.7	37.36	14.07	31.87	13.35	22.43
Female gender, %**	60.04	63.14	60.17	59.00	55.76	60.65
Race, %*** ^,^ ^a^ ^,^ ^b^ ^,^ ^c^						
** Non-Hispanic White**	64.18	65.58	80.51	77.88	16.72	50.55
** Non-Hispanic Black**	18.26	14.66	7.91	9.72	53.13	30.55
** Hispanic**	13.39	17.04	8.19	8.79	21.16	12.73
** Other**	4.17	2.72	3.39	3.61	8.99	6.18
Married, %*** ^,^ ^b^ ^,^ ^c^	57.45	60.14	64.12	57.41	53.27	42.60
**Socioeconomic status**						
Years of education*** ^,^ ^a^ ^,^ ^b^ ^,^ ^c^	12.78 (3.26)	12.76 (3.11)	14.11 (2.39)	13.21 (2.94)	13.18 (3.17)	12.03 (3.07)
Household income*** ^,^ ^b^ ^,^ ^c^	1.84 (1.50)	1.84 (1.45)	2.66 (1.37)	2.01 (1.46)	1.85 (1.49)	1.17 (1.39)
** $0–$20,000**	22.37	23.37	9.69	20.49	25.97	45.71
** $20,001–$40,000**	23.4	24.77	15.1	23.58	21.89	24.29
** $40,001–$60,000**	15.01	16.27	12.82	15.26	12.66	9.64
** $60,001–$10,0000**	17.24	15.84	23.93	18.18	16.95	9.29
** $100,001+**	21.97	19.75	38.46	22.49	22.53	11.07
Urban (%)*** ^,^ ^a^ ^,^ ^c^	53.07	51.96	51.43	50.26	66.13	48.88
U.S.-born (%)*** ^,^ ^a^ ^,^ ^c^	86.61	83.33	92.94	91.09	77.77	87.68
**Health**						
Self-rated health*** ^,^ ^b^ ^,^ ^c^	3.05 (1.07)	3.22 (1.05)	3.37 (0.95)	3.11 (1.00)	3.02 (0.99)	2.53 (0.97)
ADL*** ^,^ ^b^ ^,^ ^c^	0.59 (1.31)	0.40 (1.02)	0.26 (0.86)	0.45 (1.07)	0.41 (1.03)	1.34 (1.66)
IADL*** ^,^ ^a^ ^,^ ^b^ ^,^ ^c^	0.28 (0.79)	0.17 (0.57)	0.10 (0.45)	0.19 (0.58)	0.16 (0.58)	0.46 (0.86)
Neuroticism*** ^,^ ^b^ ^,^ ^c^	1.97 (0.62)	1.77 (0.58)	2.00 (0.60)	2.05 (0.60)	2.01 (0.61)	2.36 (0.63)
CES-D score*** ^,^ ^b^ ^,^ ^c^	1.54 (2.03)	1.01 (1.58)	1.18 (1.87)	1.47 (1.98)	1.68 (2.08)	2.81 (2.46)
Loneliness*** ^,^ ^b^ ^,^ ^c^	1.55 (0.44)	1.40 (0.39)	1.52 (0.45)	1.60 (0.44)	1.64 (0.48)	1.85 (0.43)

*Note*. ADL = activities of daily living; CES-D = Center for Epidemiologic Studies Depression Scale; IADL = instrumental activities of daily living. For group-wise comparison:

a = significant difference between Class 3 versus Class 4;

b = significant difference between Class 3 versus Class 5;

c = significant difference between Class 4 and Class 5.

*** *p* < .001.

** *p < *.01.

Characteristics varied across latent classes. Among the five latent classes, C1 (few discrimination experiences) has the largest percentage of individuals aged 75 and older, with education levels and racial/ethnic composition similar to the overall sample. It also has the lowest number of depressive symptoms, the lowest neuroticism score, and the lowest level of loneliness. C2 (discrimination with no specified attributes) appears to be the most privileged, with the highest percentage of non-Hispanic Whites (80.51%), mostly U.S.-born individuals (92.94%), the greatest levels of education and household income, the best self-rated health status, and the lowest ADL and IADL difficulties. C3 (discrimination due to ageism) is also predominantly non-Hispanic White (77.88%) and U.S.-born (91.09%), with an age composition and marital status comparable to the overall sample.

Given that C4 (discrimination due to ageism, racism, and ethnicism) and C5 (discrimination due to ageism, explicit physical characteristics, and socioeconomic disadvantages) identified co-occurring discrimination tied with sociodemographic, physical, or financial characteristics in addition to ageism (C3), group comparisons were conducted. These three groups significantly differ in the composition of age, race, ethnicity, education, and IADL (as shown in Table 2). C3 stands out with a larger proportion of respondents (31.87%) aged 75+. C4 is more racially and ethnically diverse, with a higher concentration of non-Hispanic Black (53.13%), Hispanic (21.16%), and immigrants (22.23%). C5 is the most socioeconomically disadvantaged, having the lowest marriage rate (42.60%), educational level, annual household income, and self-rated health. It also has the highest percentage of rural residents (51.12%), the most depressive symptoms, the highest neuroticism score, and the highest ADLs and IADLs.

### Discrimination attributions and mental health

Table 4 summarizes the regression results of discrimination attribution latent classes on mental health (Model 1 for depressive symptoms and Model 2 for loneliness), controlling for demographic, socio-economic, and health-related variables. We used C3 (discrimination due to ageism) as the reference group. Compared to individuals in C3, respondents in C1 (few discrimination experiences) reported significantly fewer depressive symptoms (*b *= −0.13, *p* < .000) and decreased level of loneliness (*b *= −0.12, *p* < .000). Still using C3 as the reference group, individuals in C5 (discrimination due to ageism, explicit physical characteristics, and socioeconomic disadvantages) reported more depressive symptoms (*b *= 0.41, *p* < .000) and an increased level of loneliness (*b *= 0.09, *p* < .000). C4 (discrimination due to ageism and racism/ethnicism) did not differ significantly from C3 on any of the health outcomes.

**Table 4.** Discrimination typologies and mental health outcomes (coefficients reported).

Variables	Model 1 DV: Depressive symptoms	Model 2 DV: Loneliness
**Membership (ref: C3)**		
** C1**	−0.13**	−0.12***
	(−0.22, −0.04)	(−0.15, −0.10)
** C2**	−0.08	−0.03
	(−0.26, 0.10)	(−0.07, 0.02)
** C4**	0.12	0.02
	(−0.04, 0.29)	(−0.02, 0.07)
** C5**	0.41***	0.09***
	(0.20, 0.61)	(0.04, 0.14)
**Age (ref: 50–64)**		
** 65–74**	−0.15**	−0.02
	(−0.26, −0.05)	(−0.04, 0.01)
** ≥75**	−0.28***	−0.02
	(−0.38, −0.17)	(−0.05, 0.00)
**Female gender**	0.14**	−0.08***
	(0.05, 0.22)	(−0.10, −0.06)
**Race (ref: White)**		
** Black**	0.17**	0.02
	(0.05, 0.29)	(−0.01, 0.05)
** Hispanic**	0.06	−0.00
	(−0.10, 0.22)	(−0.04, 0.04)
** Other**	0.30**	0.09***
	(0.08, 0.51)	(0.04, 0.15)
**Married**	−0.38***	−0.10***
	(−0.47, −0.28)	(−0.12, −0.07)
**Years of education**	−0.01+	−0.00*
	(−0.03, 0.00)	(−0.01, −0.00)
**Household income**	−0.08***	−0.02***
	(−0.11, −0.04)	(−0.03, −0.01)
**Urban**	−0.01	−0.03**
	(−0.10, 0.07)	(−0.05, −0.01)
**U.S.-born**	0.02	0.00
	(−0.13, 0.17)	(−0.04, 0.04)
**Self-rated health**	−0.37***	−0.04***
	(−0.42, −0.32)	(−0.05, −0.03)
**ADL**	0.23***	0.01*
	(0.18, 0.28)	(0.00, 0.03)
**IADL**	0.27***	0.02^+^
	(0.18, 0.36)	(−0.00, 0.04)
**Neuroticism**	0.91***	0.24***
	(0.84, 0.98)	(0.22, 0.25)

*Note*. ADL = activities of daily living; DV = dependent variable; IADL = instrumental activities of daily living; ref = reference group.

*** *p < *0.001.

** *p < *0.01.

* *p < *0.05.

+ *p < *0.01.

**Figure 1.**
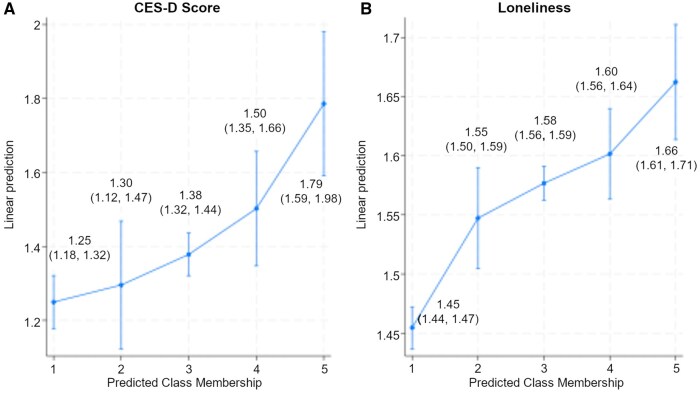
Predicted probabilities and confidence intervals of health outcomes by class membership of discrimination attributes. A higher CES-D score indicates more depressive symptoms (A), and a higher loneliness score indicates a greater level of loneliness (B). Marginal effects are the predicted changes in health outcomes associated with each latent class, holding covariates at their mean values. The non-overlapping confidence intervals (CIs) between classes indicate significant differences in their predicted outcomes, whereas overlapping CIs suggest non-significant differences between the classes. For example, C5 had significantly higher depressive symptoms than C1 to C3. CES-D = Center for Epidemiologic Studies Depression Scale.

Marginal effects at the mean were calculated to better understand how changes in discrimination typologies are associated with mental health, holding other variables constant at mean values. Figure 1 shows the predicted value of depressive symptoms (left) and loneliness (right) for each latent class, based on the mean values of covariates. Overall, Figure 1A demonstrates that depressive symptoms tend to increase as experiences of discrimination become more complex and intersectional, progressing from C1 to C5. Individuals in C5 (discrimination due to ageism, explicit physical characteristics, and socioeconomic disadvantages) reported significantly greater numbers of depressive symptoms of 1.79 (CI = 1.59, 1.98) than those in C1–C3. Similar patterns were observed regarding loneliness. As shown in Figure 1B, individuals in C5 reported higher loneliness scores of 1.66 (CI = 1.61, 1.71) than those in C1–C3.

## Discussion

The findings of this study contribute to a growing understanding of how older adults experience and attribute everyday discrimination, revealing distinct patterns that connect these experiences to mental health in later life. Through LCA, five distinct groups were identified, with ageism emerging as a prominent theme. Notably, the present study identified a large proportion of U.S. older adults who attributed their experiences of discrimination primarily to ageism (48%)—the largest among the five classes—with an additional 8% identifying ageism alongside racism and ethnicism, and 5% citing it in combination with other characteristics. Altogether, over three-fifths of respondents reported age-related discrimination, aligning with prior research highlighting its widespread prevalence among older adults. Based on existing literature, ageism is among the most common forms of discrimination among older adults (Ludwig et al., 2025). Analysis of HRS data revealed 30% of participants aged 50+ cited age as the primary reason for experiencing perceived discrimination in their day-to-day life (Luo et al., 2012). Comparable figures include 33.3% of participants aged 52+ (English Longitudinal Study of Aging; Rippon et al., 2014) and more than one in three Europeans aged 65+ (European Social Survey; Abrams et al., 2011). Though understudied, the relative dominance of age-based discrimination compared to other forms of discrimination (e.g., gender, ethnicity) has been consistently reported (Abrams et al., 2011; Ludwig et al., 2025; North & Fiske, 2015). Notably, existing literature also documented how the constellations of discrimination attributions shift when stratified by race/ethnicity (Gonzales et al., 2021). For example, Black respondents in the HRS identified race as the top reason for everyday discrimination, then age, gender, and ancestry as co-occurring attributes among pre-retirees. In contrast, Hispanic respondents and White respondents identified age as the main reason; the remaining co-occurring attributes were then ranked differently: Hispanic respondents ranked race as the second reason, then ancestry, physical appearance, and gender. White respondents attributed everyday discrimination overwhelmingly because of age, followed by gender, weight, physical appearance, physical disability, and race, being the very last reason. These findings highlight that while ageism is a common experience among older adults, the constellation of attributions varies across racial and ethnic groups, reflecting unique perspectives from minoritized populations, given the social positioning of resources, power, and privilege.

Findings confirm the intersectionality of discrimination, where ageism often co-occurs with other biases, particularly racism, ethnic discrimination, weight bias, ableism, and classism. This aligns with Crenshaw’s intersectionality theory (Crenshaw, 1989), which asserts that multiple aspects of a person’s social identity intersect to create unique experiences of discrimination and privilege. This also aligns with research from Ayalon’s team (e.g., Das & Ayalon, 2025; Ayalon & Tesch-Römer, 2018), which underscores that ageism often intersects with other forms of discrimination, such as those related to physical ability or immigration status, creating multiple jeopardies and compounded vulnerabilities, exemplified by poorer health outcomes. Such intersecting vulnerabilities highlight the importance of viewing ageism in context, as part of a broader landscape of potential discrimination, including healthism, ableism, lookism, and sexism, which target qualities other than age.

This interwoven nature of discrimination experiences emphasizes that focusing solely on one form of bias may overlook the compounded challenges faced by individuals situated at these intersections, such as respondents in categories C4 (ageism intersecting with racism and ethnicism) and C5 (ageism intersecting with weight, physical disability, appearance, or financial status). The comparison of sample characteristics across discrimination typologies supports the assumptions posited by several theoretical frameworks (Aneshensel, 1992; Pearlin et al., 1981), which suggest that discrimination exposure is structured by one’s social status. Accordingly, individuals in disadvantaged groups, such as those with lower income, racial or ethnic minority status, or physical disability, experience discrimination more frequently. As for the associations between discrimination and health domains, the results highlight the differential mental health associations with discrimination attribution typologies. As the most accepted “ism” in our society (Morrow-Howell & Gonzales, 2020), ageism is not only prevalent but also exerts deleterious effects on individuals’ well-being (Beatty et al., 2014; Chang et al., 2020; Cobb et al., 2022). As Ayalon and Tesch-Römer (2017, 2018) argued, ageism contributes to mental health disparities by fostering internalized negative stereotypes, which can lead to heightened stress and reduced psychological resilience. This perspective complements our findings that respondents who experienced ageism (C3) and ageism combined with other biases (C4 and C5) reported significantly more depressive symptoms and a higher level of loneliness compared to those with few discrimination experiences (C1), implying the toll of ageism on mental health. Ageism not only worsens mental health directly but also influences health indirectly by reducing access to healthcare, employment, and social participation (Mikton et al., 2021); the longitudinal and causal pathways merit further attention in future research.

As for those who faced discrimination due to ageism, explicit characteristics, and socioeconomic disadvantages (C5), they reported worse depressive symptoms and elevated loneliness in comparison with C3, indicating the compounded effects of multiple forms of discrimination while controlling for important covariates. This finding aligns with previous research suggesting that individuals facing multiple forms of discrimination may suffer compounded negative health outcomes due to the cumulative burden of stress and stigmatization (Cobb et al., 2022). In contrast, individuals who experienced discrimination due to ageism, racism, and ethnicism (C4) showed no significant differences from C3 across the outcomes. The lack of significant differences in health outcomes between C3 (discrimination due to ageism) and C4 (discrimination due to ageism, racism, and ethnicism) suggests that ageism may be a particularly salient stressor in later life, potentially overshadowing the incremental effects of racism and ethnicism in this sample. This finding tempers our claims about the role of intersectionality for C4, as the intersectional effects of racism and ethnicism do not appear to add a substantial additional burden beyond ­ageism. One explanation may be the unique nature of ageism: unlike racism or sexism, which typically targets individuals whose characteristics remain consistent throughout life (Ayalon & Tesch-Römer, 2017), ageism emerges as a relatively new and evolving experience as individuals transition into a marginalized social status, making it particularly stressful in older adulthood (Chang et al., 2020). Differences in group composition may also contribute. C4 participants, who are more racially and ethnically diverse (53.13% non-Hispanic Black, 21.16% Hispanic, and 22.23% immigrants) than the predominantly non-Hispanic White C3 group (77.88%), may have developed resilience and coping mechanisms for race-/ethnicity-based discrimination over their life course. However, the pronounced negative health associations in C5 (discrimination due to ageism and multiple characteristics) highlight the compounded impact of intersectional discrimination when ageism co-occurs with physical and socioeconomic attributes. These findings also underscore the need for further research to explore intersectional discrimination using measures that capture structural and institutional forms of bias, such as workplace discrimination, healthcare inequalities, or neighborhood quality, which may uncover additional dimensions of intersectionality not captured in this study. This approach aligns with Ayalon & Tesch-Römer’s (2017) call to investigate structural and institutional forms of ageism, particularly in settings such as healthcare and employment.

For instance, this study did not include experiences of work discrimination, the quality of neighborhoods, or experiences of major lifetime discrimination, such as confrontational experiences with police officers, all of which racial and ethnic minorities report at higher rates when compared to White respondents and have been shown to be associated with many health domains (Gonzales et al., 2021). The quality of education and work environments in early and midlife are associated with health outcomes in later life, with further evidence of minorities being concentrated in lower-quality schooling and work when compared to White respondents (Ahonen et al., 2018; Lövdén et al., 2020; Sheftel et al., 2025). This is another important limitation that warrants further research, especially with LCA. Nonetheless, the lack of significant differences between C3 and C4 does not negate the importance of intersectional discrimination but rather highlights the need to understand the unique role of ageism within intersectional frameworks. These findings underscore nuanced typologies in which different types of interpersonal discrimination are associated with health, with multiple forms of bias being consequential to mental health.

The differential health associations across classes are noteworthy. While discrimination was generally associated with poorer health, the negative associations were most pronounced for respondents who experienced discrimination related to intersectional factors. The marginal effects analysis provides further insights into how different discrimination attribution typologies are associated with mental health, with all other variables held constant at their mean values. Distinct patterns emerge across depressive symptoms and loneliness. In Figure 1A and B, the marginal effect plots demonstrate the associations of discrimination with depression and loneliness. The results indicate that individuals who attributed discrimination to ageism combined with racism/ethnicism (C4) or explicit characteristics (C5) reported heightened levels of depression and loneliness while controlling for important covariates. The results provide further support for cumulative (dis)advantage theory and are consistent with the stress process model (Pearlin et al., 1981), social stress theory (Aneshensel, 1992), and minority stress (Forrester et al., 2019), all of which posit that interpersonal discrimination acts as a stressor, triggering chronic stress responses that undermine mental health. In summary, these marginal effects highlight the compounded negative associations of discrimination, especially when multiple forms of bias intersect and are linked with these mental health indicators. This finding also suggests that certain types of discrimination may carry a heavier toll on well-being due to the added layers of social stigma and marginalization. Future research should incorporate variables such as stigma severity to account for their potential impact.

These results have important implications for interventions and policies aimed at reducing ageism and other forms of discrimination. Given the high prevalence and significant health toll of ageism, combating it has been identified as one of the four key action areas of the Decade of Healthy Ageing (2021–2023) (United Nations, 2020). Feasible, low-cost intervention strategies, such as education and intergenerational contact, have been shown to be effective in reducing ageism (Burnes et al., 2019). Moreover, due to ageism’s intersection with other forms of discrimination, it is critical to develop targeted strategies that address both the age-specific and intersectional nature of discrimination. Healthcare providers should be particularly mindful of the diverse discrimination experiences of older adults and how these experiences may impact health, especially for individuals from marginalized or intersectional groups. Programs designed to reduce ageism, while also recognizing the compounded challenges faced by individuals who experience multiple sources of bias, may be more effective in mitigating the adverse mental health effects observed in this study.

The use of LCA in this study offers several strengths over simpler methods, such as counting the number of reported discrimination attributions. LCA is a person-centered approach that identifies latent subgroups based on patterns of co-occurring attributions without relying on predefined categories. This method is particularly suited for capturing the intersectional nature of discrimination, allowing us to uncover nuanced typologies that reflect intersecting biases, such as ageism with racism/ethnicism or physical/socioeconomic characteristics. The rigorous use of model fit statistics (e.g., AIC, BIC, entropy) and classification diagnostics (e.g., AvePP, OCC) ensured a statistically robust and interpretable five-class solution. However, LCA has limitations that should be considered. Selecting the optimal number of classes involves balancing statistical fit with interpretability, introducing some subjectivity despite the use of objective criteria. Furthermore, while comprehensive, the reliance on HRS discrimination attribution items may not capture all forms of discrimination (e.g., structural or institutional), potentially limiting the scope of typologies. Finally, the cross-sectional design limits our ability to assess the temporal stability of these classes, which could be explored in future longitudinal studies.

The study’s limitations should be considered while interpreting the results. First, the Everyday Discrimination Scale used in the HRS may be cognitively demanding for some respondents, potentially contributing to missing responses in the intensity question, which asks about the frequency of discrimination experiences. A solution would be to modify the scale’s sequencing by first asking respondents if they have experienced any form of bias (yes/no), followed by identifying specific attributions, and then assessing the intensity of unfairness for each attribution to better capture the nuanced nature of perceived discrimination. This restructuring could reduce respondent burden and improve data completeness. Relatedly, the question of the everyday discrimination attribution items does not specify the extent of severity of exposure to specific sources of discrimination. In the LCA, these items were treated with equal weight, even though discrimination of different sources may vary in intensity and time frame. Relatedly, the dataset’s lack of certain information makes it challenging to explore different forms of discrimination, such as overt (or blatant) versus covert (or subtle) discrimination, or institutional and structural versus intra- and interpersonal discrimination. Second, given the complexity and fluid nature of social identity, the cross-sectional study design is insufficient to explore how discrimination attributions change over time and across the life course. While such a design effectively captures a snapshot of discrimination attribution typologies, it limits the ability to examine how these evolving factors contribute to the mental health outcomes of older adults over time. It also precludes establishing causal or bidirectional relationships between discrimination attributions and health outcomes and cannot adequately control for potential confounders such as neuroticism that may influence both attributions and outcomes. A longitudinal approach with directionality testing and controls for additional confounders would provide more robust insights into these dynamics. Additionally, the HRS dataset’s focus on adults aged 50 and older and its limited racial and gender diversity, with only three racial categories and minimal non-binary representation, restricts the ability to fully capture the intersectional experiences of discrimination across diverse populations. Future research should consider including more diverse age groups and expanding racial and gender representation to better understand how discrimination attributions vary across the lifespan and different population groups. Finally, the “other” racial and ethnic category should be disaggregated and analyzed, as emerging research has shown disproportionately high levels of perceived everyday discrimination and its strong association with cognitive health among Indigenous and Asian American older adults (Takamura et al., 2022; Whetung, 2023).

In conclusion, this study underscores the importance of considering attributions of discrimination in relation to older adults’ mental health. Ageism, as a predominant form of discrimination, often intersects with other forms of bias, leading to disproportionately negative health for individuals facing multiple sources of stigmatization. Addressing these disparities requires a multifaceted approach that acknowledges the complex and intersecting nature of discrimination in later life.

## Data Availability

Data were drawn from the 2016 Health and Retirement Study (https://hrs.isr.umich.edu). The authors decided not to preregister this study to share data and analytic code because the researchers have not completed the planned analyses for future publications.
